# Molecular epidemiology and genetic diversity of *Listeria monocytogenes* isolates from a wide variety of ready-to-eat foods and their relationship to clinical strains from listeriosis outbreaks in Chile

**DOI:** 10.3389/fmicb.2015.00384

**Published:** 2015-04-30

**Authors:** David Montero, Marcia Bodero, Guillermina Riveros, Lisette Lapierre, Aldo Gaggero, Roberto M. Vidal, Maricel Vidal

**Affiliations:** ^1^Programa de Microbiologia y Micología, Instituto de Ciencias Biomédicas, Facultad de Medicina, Universidad de ChileSantiago, Chile; ^2^Laboratorio de Salud Pública Ambiental y Laboral, Secretaría Regional Ministerial de Salud Región MetropolitanaSantiago, Chile; ^3^Facultad de Ciencias Veterinarias y Pecuarias, Universidad de ChileSantiago, Chile; ^4^Programa de Virología, Instituto de Ciencias Biomédicas, Facultad de Medicina, Universidad de ChileSantiago, Chile

**Keywords:** *L. monocytogenes*, ready-to-eat food, outbreaks, PFGE, clonal relationship

## Abstract

*Listeria monocytogenes* is a pathogen transmitted through food that can cause severe infections in high-risk groups such as pregnant women, elderly, young children and immunocompromised individuals. It is a ubiquitous bacterium that can survive in harsh conditions, such as dry environments, at low temperatures, in brine conditions and at low pH values. It also has the capacity to form biofilms, which makes it particularly successful even in colonizing surfaces within food processing plants. This study analyzed the presence of *L. monocytogenes* in ready-to-eat food (RTE) such as sausage, cheese, fresh salads, and other types of raw food. 850 samples of refrigerated and packaged food collected in 2008 and 2009 were analyzed. It was found that 25% of these samples were contaminated with *L. monocytogenes* strains. Serotyping and virulence genes detection by polymerase chain reaction (PCR) identified that strains belonging to serotype 4b, and containing one or more genes encoded by pathogenicity island (LIPI-1), were significantly associated with specific food types. Furthermore, using pulse field gel electrophoresis (PFGE), it was possible to associate isolates from cheese with strains from clinical cases of listeriosis outbreaks that occurred during the same time period within the same geographic regions. In addition, a strong correlation was observed between isolates from frozen seafood and from clinical strains obtained from sporadic cases of listeriosis. In agreement with reports described in other countries, our results shown that Chilean strains of *L. monocytogenes* from food products include the most virulent serotypes, encoding for the main virulence genes of the LIPI-1, and were clonally related to clinical isolates from sporadic cases and outbreaks of listeriosis. In conclusion, we show that Chilean isolates of *L. monocytogenes* from RTE and raw food products can cause disease in humans, representing a public health risk that justifies permanent surveillance.

## Introduction

*Listeria monocytogenes* is a pathogen transmitted by food products that may cause listeriosis, a severe infection in humans that has been recognized around the world as a serious public health problem (Farber and Peterkin, 1991; Swaminathan and Gerner-Smidt, 2007). This disease may present either in its invasive form or as febrile gastroenteritis (Vázquez-Boland et al., 2001; Franciosa et al., 2005). The invasive form, which has a high mortality rate (20–30%), principally affects the elderly and immunocompromised individuals, who may have different clinical presentations such as septicemia, meningitis and meningoencephalitis. In nursing infants and pregnant women the invasive form can cause perinatal infections and spontaneous abortions, respectively (Schuchat et al., 1991; Vázquez-Boland et al., 2001; Siegman-Igra et al., 2002). In contrast, febrile gastroenteritis caused by this pathogen is a self-limiting infection that generally affects healthy individuals after ingesting a large inoculum of this bacterium from contaminated food products (Ooi and Lorber, 2005).

*L. monocytogenes* is widely disseminated in the environment due to its ability to survive for long periods of time in adverse conditions, such as in dry environments, at low temperatures, in brine conditions and at a wide range of pH values (Weis and Seeliger, 1975; Fenlon et al., 1996; Gandhi and Chikindas, 2007). In addition, this pathogen produces biofilms and colonizes and persists in food processing plants, making it a threat for both food industry and consumers (Carpentier and Cerf, 2011). There is heterogeneity in the virulence of *L. monocytogenes* isolates, and there is no clear correlation between the potential for virulence and the origin of a strain. However, out of the 13 serotypes described for the species, 1/2a, 1/2b, and 1/2c are frequently isolated from food products, of which serotypes 1/2a, 1/2b, and 4b cause 95% of the human cases of listeriosis. Most listeriosis outbreaks are associated with strains of serotype 4b (Swaminathan and Gerner-Smidt, 2007).

Epidemiological monitoring studies of *L. monocytogenes* have used different genotyping techniques (Chen et al., 2010, 2011, 2014; Parisi et al., 2010; Lambertz et al., 2013). However, pulse field gel electrophoresis (PFGE), to date, has been the method with the greatest discrimination and reproducibility for molecular sub-typing of this pathogen and is considered the gold standard (Gerner-Smidt et al., 2006; Chenal-Francisque et al., 2013). Also, epidemiological studies of *L. monocytogenes* with PFGE generally involve serotype analysis (Gianfranceschi et al., 2009; Korsak et al., 2012; Lambertz et al., 2013) and the identification of virulence genes (Lomonaco et al., 2012). Genotyping and comparison of bacterial isolates from both food samples and clinical cases over the same time period and in the same geographic region may reveal possible sources of contamination, which would allow the identification and regulation (of production, distribution, and consumption) of foods that represent a public health risk.

There has been little work done to characterize infections due to *L. monocytogenes* in Chile. Additionally, there have only been a few studies on the incidence of this pathogen in food (Sedano et al., 2013). Prior to 2008, only sporadic cases of listeriosis had been reported in Chile, with an incidence rate of three cases per million people. However, in 2008 there was a massive outbreak with 165 reported cases and 14 deaths, which was associated with the consumption of two types of soft cheese: Brie and Camembert. This was the first outbreak in Chile with a clear epidemiological association between the consumption of a particular type of food and listeriosis (MINSAL, 2009). Another listeriosis outbreak occurred in 2009, which was related to the consumption of sausage and other meat products, with a total of 73 reported cases and 17 deaths (MINSAL, 2009). Thus, the recent emergence of this pathogen in Chile, despite implementation of surveillance measures and food legislation, is clear. The main objectives of this study were to demonstrate the presence of *L. monocytogenes* in RTE and raw food products, to characterize the presence of virulence genes in bacterial isolates and establish genetic relationships between food isolates, and clinical strains isolated from listeriosis cases in Chile.

## Materials and methods

### Food samples

We analyzed 850 food samples taken from different retail stores and factories in Santiago, which were selected based on the prevalence of *L. monocytogenes* in previous national and international studies (Cordano and Rocourt, 2001; Cordano and Jacquet, 2009). A summary of the type and number of samples analyzed is provided in Table 1.

**Table 1:** **Serotypes and genetic determinants of virulence of *Listeria monocytogenes* isolates**.

**Food type**	**N**	**No. of positive samples (%)**	**No. of isolates**	**Serotype No. of isolates (%)**					**Genetic determinants of virulence *No. of isolates (%)***	
				**1/2a**	**1/2b**	**1/2c**	**4b**	**NT**	LIPI-1^*^	**LIPI-1 +*inlAB***
Raw meat	85	21 (25)	21	8 (38)	1 (5)	3 (14)^a^^(+)^	4 (19)^b^^(−)^	5 (24)^c^^(+)^	7 (33)	7 (33)
Raw poultry	85	10 (12)	10	6 (60)	2 (20)	1 (10)	1 (10) ^a^^(−)^	0	2 (20) ^a^^(−)^	2 (20)
Cooked sausage	41	5 (13)	5	1 (20)	4 (80)^c^^(+)^	0	0	0	1 (20)	1 (20)
Frozen seafood	197	50 (25)	38	10 (26)	3 (8)	0	25(66)^a^^(+)^	0	23 (60)	23 (60)
Pâté	62	34 (55)	32	20 (62)^c^^(+)^	3 (9)	1 (3)	4 (13)^c^^(−)^	4 (13)	5 (15)^c^^(−)^	5 (15)^c^^(−)^
Smoked fish	69	18 (26)	16	5 (31)	7 (44)^c^^(+)^	0	3 (19)^a^^(−)^	1 (6)	14 (88)^b^^(+)^	14 (88)^b^^(+)^
Cheese	116	46 (40)	46	0	0	0	46 (100)	0	41 (89)^c^^(+)^	32 (70)^c^^(+)^
Frozen vegetables	90	26 (29)	25	12 (48)	3 (12)	2 (8)	8 (32)	0	10 (40)	9 (36)
Fresh vegetables	105	2 (2)	2	0	2 (100)	0	0	0	0	0
Total	850	212 (25)	195	62 (32)	25 (13)	7 (4)	91 (46)	10 (5)	103 (53)	93 (48)
Clinical isolates			40	9 (23)	10 (25)	0	21 (52)	0	26 (65)	24 (60)

*N, Number of samples analyzed; NT, Not typeable*.

* *Positive isolates of all genes present in the Listeria pathogenicity island 1 (LIPI-1), i.e., prfA, plcA, hly, mpl, actA, and plcB*.

(−) *Prevalence significantly lower compared to all other food isolates*.

(+) *Prevalence significantly higher compared to all other food isolates*.

a, b, c *P-value for the association between the specific food vs. all other food isolates and the specified serotype/genetic determinant of virulence vs. all other serotypes/genetic determinants of virulence (^a^P < 0.05; ^b^P < 0.01; ^c^P < 0.001)*.

*The underlined values means that only serotype isolated in respective food category*.

### Isolation and identification of *Listeria monocytogenes*

Bacterial detection in food samples was performed using the VIDAS *Listeria* DUO kit (Biomerieux), according to the manufacturer's instructions. Briefly, analytical portions of 25 g of each sample were pre-enriched for *Listeria* in 225 ml of *Listeria* Xpress (LX) Broth and incubated at 30°C for 22–24 h. Then 0.1 ml of this broth was transferred to 6 ml of LX Broth and again incubated at 30°C for 22–24 h. Finally, 500 μl of each sample were tested in the VIDAS platform. Positive samples according to the VIDAS test were seeded in ALOA chromogenic agar (Agar *Listeria* Ottaviani and Agosti, Biomerieux), and further confirmed by the API *Listeria* kit (Biomerieux) and seeding in TSA agar + 5% sheep blood. Confirmed colonies were conserved in 20% non-fat milk at −20°C and 40% glycerol at −70°C. The clinical strains were kindly provided by the Instituto de Salud Pública de Chile. All strains were collected from patients with listeriosis in the years 2008 and 2009. The number of isolated strains from food and clinical samples are shown in Table 1. The project protocols were approved by the Comite Asesor de Bioética de FONDECYT—CONICYT, Chile.

### Identification of serotypes and virulence genes by PCR

The identification of *L. monocytogenes* virulence genes was performed by PCR amplification of the genes that form the LIPI-1 pathogenicity island (*prfA, plcA, hly, plcB, mpl*, and *actA*) and the genes *inlA* and *inlB*, which code for internalin proteins A and B, respectively. Primers were designed using at least three sequences of each gene available in GenBank and by obtaining a region of homology, which was then analyzed with the Primer3 software. All primers were designed in-house and are described in Table 2. To determine the *L. monocytogenes* serotypes we used primers described by Doumith et al. (2004). DNA was extracted from colonies grown in trypticase soy agar with yeast extract (TSAYe), which were boiled in 150 μl of bi-distilled sterile water twice for 10 min and centrifuged; 2 μl of the supernatant was used as template for the PCR reaction. The reaction mixture for the LIPI-1 island and internaline genes contained primers at a final concentration of 2 mM, 0.65 U Taq DNA Polymerase, 0.2 mM dNTPs, 1X reaction buffer and 1.5 mM MgCl2 in 25 μl final volume. For serotyping these components were used in the same concentrations, except for the primers, which were 1.5 mM lmo1118, 1 mM lmo0737, ORF 2819, and ORF2110 and 0.2 mM PrsF and PrsR. The control strains used for the PCR reactions were: *L. monocytogenes* ATCC 19115, *L. ivanovii* ATCC 19119 and four isolates obtained from food analyzed in this study, *L. innocua* (frozen shellfish), *L. grayi* (raw poultry), *L. welshimeri* (raw meat) and *L. seeligeri* (smoked fish). The amplification program for each type of gene is provided in the Supplementary Table S1.

**Table 2:** **Primers used in amplification of *L. monocytogenes* virulence genes**.

**Primer**	**5′-3′ Sequence**	**Tm (°C)**	**Protein coded by target gene (gene)**	**Size (pb)**
prfA-F prfA-R	ACCAATGGGATCCACAAGAA GCTTCCCGTTAATCGAAAAAT	58	Transcripcional regulator A (*prf*A)	330
lcA-F plcA-R	TCCCATTAGGTGGAAAAGCA CGGGGAAGTCCATGATTAGA	57	Phosphatidyl inositol Phospholipase C (*plc*A)	840
Hly-F Hly-R	GTCTACCAATTGCGCAACAA TGGTGTTTCCCGGTTAAAAG	57	Listeriolysine O (*hly*)	1100
Mpl-F Mpl-R	AAAGGTGGAGAAATTGATTCG AGTGATCGTATTGTAGGCTGCTT	62	Metaloprotease (*mpl*)	450
actA-F actA-R	AAACAGAAGAGCAGCCAAGC TTCACTTCGGGATTTTCGTC	58	Protein for actin nucleation (*act*A)	571
plcB-F plcB-R	CAGCTCCGCATGATATTGAC CTGCCAAAGTTTGCTGTGAA	58	Phosphatidyl choline phospho-lipase C (*plc*B)	723
Inl 1A-F Inl 1A-R	GGCTGGGCATAACCAAATTA CTTTTGTTGGTGCCGTAGGT	60	Internalin A (*inl*A)	629
Inl 1B-F Inl 1B-R	CCTAAACCTCCGACCAAACA CCATTTCGCGCTTCTCTATC	60	Internalin B (*inl*B)	293
prfA3-F prfA2-R	AACGGGATAAAACCAAAACAA CTATGTGCGATACCGCTTGA	58	Transcripcional regulator A (*prf*A)	506
Hly2-F Hly2-R	TCTACCAATTGCGCAACAAA GCAGGAGGATTTTCTGCATT	57	Listeriolysine O (*hly*)	852

### DNA macrorestriction and pulse field gel electrophoresis (PFGE)

The PFGE protocol was performed as previously described by Graves and Swaminathan (2001). Briefly, bacterial suspensions adjusted to optical density of 1.3 at 610 nm were embebbed in 1.2% SeaKem Gold agarose plugs. The lysed, washed five times and a 2 mm thick piece was cut, equilibrated and digested with 50 U of *ApaI* enzyme at 37°C for 5 h. The macrorestriction fragments were separated by electrophoresis on a CHEF-DR III BIO-RAD in a 1% Pulse Field Certified Agarose gels (Ultrapure DNA grade agarose) at 6 V/cm and 14°C by 21 h. We used a strain *Salmonella* serotype Braenderup H9812, previously digested with *XbaI* as a base pair marker and a run control. Images were analyzed with the software BioNumerics GelCompar II 6.0 (Applied Maths, Sint-Martens-Latem, Belgium). The similarity between fingerprints was determined using Dice's correlation coefficient with a 1% tolerance between band positions. We defined pulsotypes and pulsogroups with similarities of ≥95 and 80%, respectively. The cluster analysis and generation of dendrograms was performed using UPGMA.

### Statistical analysis

Associations between categorical variables (food category, serotype, genetic determinants of virulence and pulsogroups) were analyzed using Pearson's Chi-squared test, or when appropriate, Fisher's exact test. A value of *P* ≤ 0.05 was considered statistically significant.

## Results and discussion

Infections by *L. monocytogenes* have a low incidence rate, but the high degree of mortality, primarily in the high-risk groups (Vázquez-Boland et al., 2001). The above support the need to perform epidemiological monitoring studies in order to identify foods contaminated with this pathogen that represent a public health risk. There are few reports on the prevalence of *L. monocytogenes* in food consumed in Chile, thus the real situation of listeriosis remains to be fully characterized in the country. In this work, we analyzed 850 samples of ready-to-eat (RTE) food and raw meat, and found that 25% of these were contaminated with *L. monocytogenes*. The results are summarized in Table 1. Several RTE food and types of raw meat have been analyzed in earlier studies in Chile. Cordano and Rocourt (2001) reported prevalence rates of 1, 12, 1.25, and 4.5% in cheese, shellfish, pâté and sausages, respectively. These values greatly differ from those obtained in our study where cheese, shellfish, pâté, and sausages showed prevalence of 40, 25, 55, and 13%, respectively. Clearly pâté (34/62) and cheese (46/116) were two food products contaminated with the greatest proportion of positive samples for *L. monocytogenes*. In another study, Cordano and Jacquet (2009) analyzed frozen and fresh vegetables from 2000 to 2005, reporting a *L. monocytogenes* prevalence of 26 and 10% of samples, respectively. Our results were quite similar, with positivity rates of 29 and 2%, respectively. These findings are important, because current Chilean law considers the surveillance of *L.monocytogenes* only in RTE food products. In addition, the zero tolerance regulation mandates that *L. monocytogenes* to be absent in food products supporting bacterial growth such as cooked sausage, cheese (except those with less than 0.9 aw) or ready to eat packed meals refrigerated for 5 or more days. In food not supporting growth such as raw sausage, ice cream, food with a high percentage of salted or smoked salmon, it is necessary to proceed with bacterial counting. In this case, current regulations state that at the end of product life the counts of *L monocytogenes* should not exceed 100 CFU/gr. It is relevant to report that during this study, the present legislation had not yet been implemented, and neither active pathogen monitoring was performed. For this reason, we were focused on determining if the pathogen was present or not in food products, coinciding our work with outbreaks of listeriosis in Chile. Currently, regulation is applied to RTE and other food based on our results.

*L.monocytogenes* strains belonging to serotypes 1/2a, 1/2b, and 4b are frequently associated to listeriosis cases (Table 1). In this regard, it was interesting to determine the serotypes associated with 195 *L. monocytogenes* strains by PCR. Ten strains isolated from food (10/195) could not be serotyped by PCR. We also determined the serotype of 40 additional strains isolated from listeriosis cases occurred in Chile in 2008 and 2009 (Table 1). Notably, all strains isolated from cheese belonged to the serotype 4b. Similarly, 66% (25/38) of the strains isolated from frozen seafood were serotype 4b, and this prevalence was significantly greater (*P* < 0.05) than for other food types. Our study revealed some associations, particularly between cheese and frozen shellfish with serotype 4b (Table 1), a significant result for public health considerations due to the epidemiological relevance of this serotype (Swaminathan and Gerner-Smidt, 2007). By contrast, the other food types (with the exception of frozen vegetables) were negatively associated with serotype 4b (*P* < 0.001). Serotype 1/2a was associated with pâté (*P* < 0.05); 1/2b with cooked sausage (*P* < 0.05), smoked fish (*P* < 0.05) and fresh vegetables; and serotype 1/2c was associated with raw meat (*P* < 0.05). While the study by Cordano and Jacquet (2009) reported an association between frozen vegetables and serotype 1/2a and fresh vegetables and serotype 1/2c, we did not find an association between frozen vegetables and any serotype, and two strains isolated from fresh vegetables belonged to serotype 1/2b. Interestingly, the percentage of *L. monocytogenes* isolates in raw meat was significantly lower than that observed in RTE foods. In addition, the serotypes characterized did not correspond with those detected in human cases of listeriosis. In contrast, the association between smoked fish and serotype 1/2b along with the fact that this food type is rarely heated, makes monitoring necessary during its production and distribution, importantly preventive/corrective measures need to be implemented in Chile and other countries producers smoked fish. Among the clinical isolates the most common serotype was 4b (21/40), although 1/2a (9/40) and 1/2b (10/40) were also present. The differences between our results and previous reports in Chile suggest that *L. monocytogenes* strains are not temporally or geographically specific. As a consequence, it is necessary generate a database of *L. monocytogenes* genotypes that allows detect temporal persistence of epidemic clones and potentially virulent strains in the country and compare it with worldwide information.

This is the first Chilean report describing virulence genes in strains of *L. monocytogenes* isolated from both food products and clinical cases of listeriosis. Several studies had shown that the identification of virulence genes in *L. monocytogenes* isolates may be associated with health risks due to the consumption of food products contaminated with potentially pathogenic strains (Chen et al., 2009; Lomonaco et al., 2012; Shen et al., 2013). The genes encoded in the pathogenicity island 1 (LIPI-1) are responsible for key processes related with their intracellular life cycle; internalins A (*inl*A) and B (*inl*B) have been proven necessary and sufficient to trigger the internalization of this pathogen by susceptible non-phagocytic cells (reviewed in Vázquez-Boland et al., 2001). Presence of genes encoding for the internalins, *inl*A and *inl*B, were detected by PCR in order to determine the pathogenic potential of the different strains. All strains isolated from food samples and listeriosis cases were positive for one or more genes encoded in LIPI-1. The presence of *inl*A was more frequent than *inl*B; some strains isolated from food samples lacked both genes, while at least one of them was present in every clinical isolate (data not shown). On the other hand, percentages of both genes were quite similar (Table 1). Evaluation of all genes encoded in LIPI-1 and internalins A and B regarding the type and origin of food products showed that these genes were more prevalent in cheese (*P* < 0.001) and smoked fish (*P* < 0.01) compared to other food types. Accordingly, these genes were present in lower frequencies in strains isolated from pâté (*P* < 0.001) and raw poultry meat (*P* < 0.05). Notably, none of the strains isolated from fresh vegetables tested positive for any of these genes. Other food types did not show a significant association with these virulence genes despite the fact that a notable proportion of strains isolated from frozen shellfish were positive. However, 65% of human strains were positive for LIPI-1 and 60% for LIPI-1 and *inlAB*, indicating that these virulence genes are frequent in *L. monocytogenes* strains that infect humans. However, the absence of one of these genes did not imply that a strain was not virulent. Interestingly, serotype 4b had a significantly higher presence of these genes (Table 3). Thus, the analysis of serotypes and the identification of virulence genes suggested that strains isolated from cheeses, smoked fish and frozen shellfish are potentially virulent. It cannot be ruled out that strains isolated from other food products could also cause illness.

**Table 3:** **Association between serotypes and virulence genes**.

**Serotype**	**No. of isolates**	**Virulence Genes No. of isolates (%)**	
		LIPI-1^*^	**LIPI-1 + *inlAB***
1/2a	71	10 (14)^c^^(−)^	9 (13)^c^^(−)^
1/2b	35	19 (54)	19 (54)
1/2c	7	0	0
4b	112	99 (88)^c^^(+)^	88 (79)^c^^(+)^
NT	10	1 (10)^b^^(−)^	1 (10)^a^^(−)^
Total	235	129 (55)	117 (50)

*NT, Not typeable*.

* *Number of isolates positive for all genes present in Listeria's pathogenicity island 1 (LIPI-1), i.e., prfA, plcA, hly, mpl, actA, and plcB*.

(−) *Prevalence significantly lower compared to all other serotypes*.

(+) *Prevalence significantly higher compared to all other serotypes*.

a,b,c *P-value for the association between the specific serotype vs. all other isolates or the specified virulence gene vs. all other virulence genes (^a^P < 0.05; ^b^P < 0.01; ^c^P < 0.001)*.

Interestingly, the virulence genes evaluated herein were prevalent in serotype 4b and infrequent in serotypes 1/2a and 1/2c, which coincides with the frequent association of serotype 4b with outbreaks and sporadic cases of listeriosis. In addition, the presence of these genes in clinical isolates demonstrates that they are common in pathogenic strains but not necessarily responsible of illness. In fact, there are reports that *L. monocytogenes* strains with low pathogenic potential harbor these genes (Jiang et al., 2006), in contrast to pathogenic strains, which *in vitro* show a virulent profile, but when they had been tested in animal models have non-functional virulence genes (Roche et al., 2009). Thus, the presence or absence of all or some of these genes does not guarantee that a strain will or will not be virulent.

**Figure 1:**
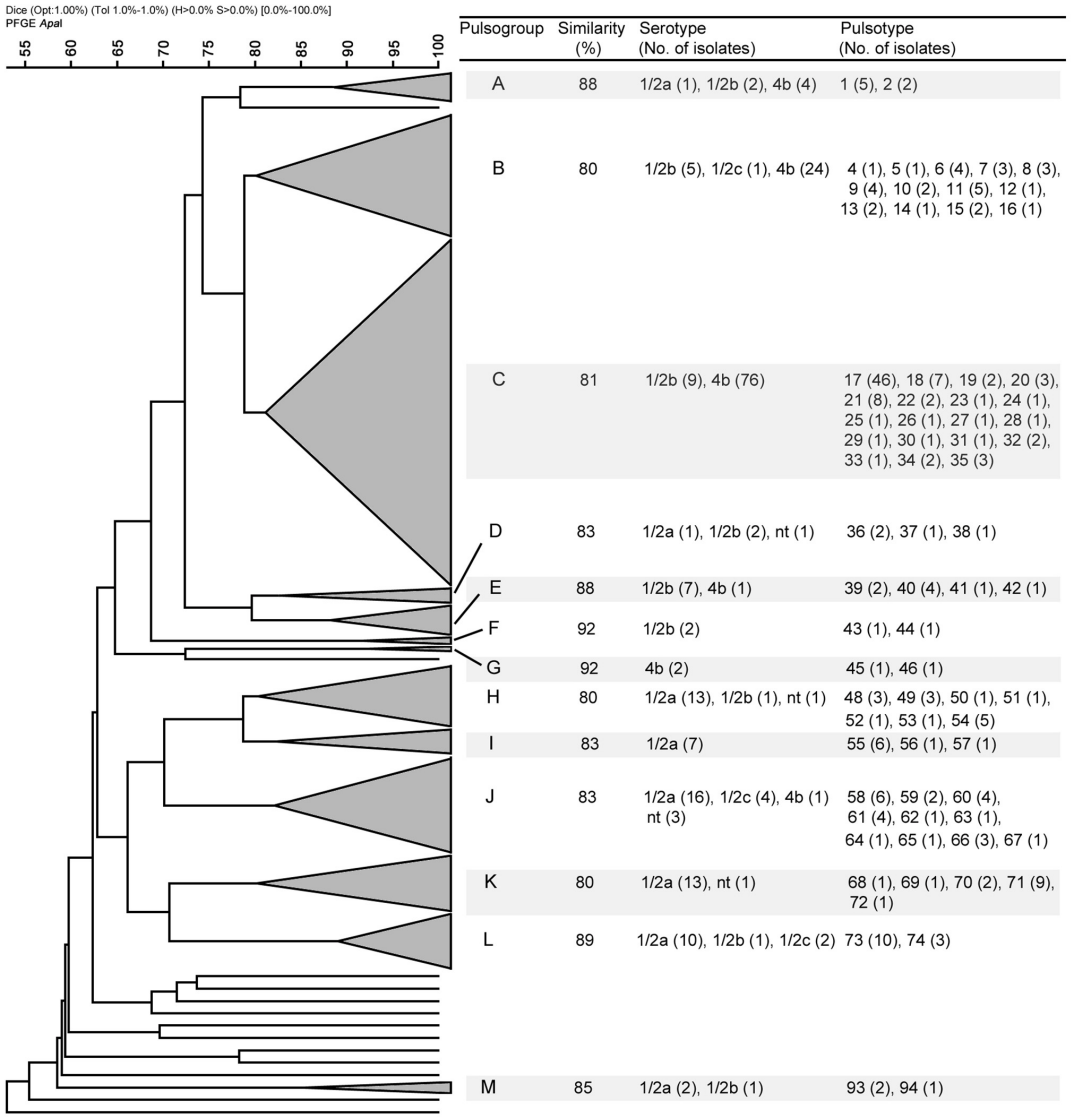
**Dendrogram showing the PFGE analysis with the most relevant characteristics of the 13 pulsogroups described (A–M)**.

Since the isolates from food samples coincide temporally and geographically with the clinical strains characterized in this study, it was possible to determine by PFGE whether the food types analyzed were possible sources of contamination. A total of 187 representative strains from food and the 40 strains of clinical origin were studied. Macrorestriction using *Apa*I provided 96 pulsotypes (numbered 1–96) that grouped into 13 pulsogroups (numbered A–M) (Table 4). Pulsogroups contained 2–19 pulsotypes, with a global similarity ≥80%. Only 13 strains, one of which was of clinical origin, were not related to a specific pulsogroup (Figure 1). The strains of the same pulsotype were generally isolated from the same food type (data not shown), and some pulsogroups were significantly associated with one or two food types (Table 4). Interestingly, pulsogroups A, B, C, and J contained 95% (38/40) of the clinical isolates and were significantly associated with pâté (*P* < 0.05), frozen shellfish (*P* < 0.001), cheeses (*P* < 0.001), and raw meat (*P* < 0.001). In addition, the most common pulsotype (17, pulsogroup C) is represented by 12 clinical strains and 33 strains isolated from cheeses (Figure 1). The results also suggest that the strains isolated from pâté, frozen shellfish, and raw bovine meat are genetically related to those causing sporadic cases of listeriosis. However, the lack of epidemiological information does not allow to establish a clear-cut association of these food products with disease. Similar studies in other countries have identified contamination sources and hazardous food types, allowing for control/monitoring of their distribution and consumption (Gilbreth et al., 2005; Gianfranceschi et al., 2009; Lambertz et al., 2013). It is important to note that clonal strains were identified in cheeses and listeriosis cases, suggesting that this was the main source of contamination in 2008 and 2009. Similarly, outbreaks with high levels of mortality and associated with cheese consumption have been reported in other countries (Linnan et al., 1988; Fretz et al., 2010). In fact, the consumption of milk and cheese is always associated with a high risk, since both products offer favorable growth conditions for almost all pathogenic bacteria (Danielsson-Tham et al., 2004). We identified other foods contaminated with strains of *L. monocytogenes* that are genetically similar to those isolated from listeriosis cases; this information underlines the need to continue monitoring these foods. The identification of clones or strains genetically similar to strains of *L. monocytogenes* that cause listeriosis cannot be taken as direct proof that a particular food was the transmission vehicle. As our results demonstrate, some pulsogroups were associated with different food types; in particular, some pulsotypes corresponded to strains isolated from different sources. This is even more relevant in the establishment of associations between food types and sporadic cases of listeriosis. Lastly, two limitations of this study were that the strains characterized might be different from the currently circulating strains, and that we did not determine the number of colony-forming units per gram recovered from food samples. Future research and the possibility of collecting new *L. monocytogenes* strains, will allow knowing whether bacterial populations continue or modify their virulence gene patterns, serotypes, or pulsotypes, helping to build an epidemiological history of *Listeria monocytogenes*.

**Table 4:** **Pulsogroups of *L. monocytogenes* isolates recovered from food samples and clinical cases in Chile (2008–2009)**.

**Pulsogroup**	**Food Types (No. of isolates)**										**No. of clinical isolates**
	**No. of isolates**	**RM**	**RP**	**CS**	**FS**	**P**	**SF**	**C**	**FV**	**V**	
A	4	0	0	1	0	3^a^^(+)^	0	0	0	0	3
B	23	2	0	0	16^c^^(+)^	1	3	0	0	1	7
C	64	2	2	0	6^a^^(−)^	0	0	45^c^^(+)^	9	0	21
D	4	0	0	0	1	2	0	0	1	0	0
E	8	0	1	0	0	2	5^c^^(+)^	0	0	0	0
F	1	0	0	0	1	0	0	0	0	0	1
G	2	0	0	0	2	0	0	0	0	0	0
H	15	0	5^c^^(+)^	1	0	6^a^^(+)^	3	0	0	0	0
I	7	0	0	0	0	7	0	0	0	0	0
J	17	10^c^^(+)^	2	0	0	4	0	0	1	0	7
K	14	1	0	0	8^b^^(+)^	2	3	0	0	0	0
L	13	0	0	0	0	0	0	0	13	0	0
M	3	0	0	2	1	0	0	0	0	0	0
Isolates without pulsogroup	12	5^b^^(+)^	0	0	1	3	2	0	0	1	1
Total	187	20	10	4	36	30	16	45	24	2	40

*RM, Raw meat; RP, Raw poultry; CS, Cooked sausage; FS, Frozen seafood; P, Pâté; SF, Smoked fish; C, Cheese; FV, Frozen Vegetables; V, Vegetables*.

(−) *Prevalence significantly lower compared to all other food isolates*.

(+) *Prevalence significantly higher compared to all other food isolates*.

a,b,c *Represent the P value of the association between the specific food vs. all other isolates and 588 the specified pulsogroup versus all other pulsogroup (^a^P < 0.05; ^b^P < 0.01; ^c^P < 0.001)*.

## Conclusion

The results obtained in this study show the presence of *L. monocytogenes* in various types of RTE food and raw meats in Chile. The prevalence of serotype 4b and the frequency of virulence genes detection in strains isolated from cheeses, frozen shellfish and smoked fish suggest a potential health risk associated with these foods. Notably, our results are consistent with reports by the Ministerio de Salud and support the association between cheese and cooked sausage consumption and the listeriosis outbreak of 2008 and 2009, respectively. Finally, this study demonstrates the need to continue performing genotyping studies on strains isolated from both food samples and clinical cases, which contribute to establishing the epidemiological situation of this emerging pathogen in Chile.

### Conflict of interest statement

The authors declare that the research was conducted in the absence of any commercial or financial relationships that could be construed as a potential conflict of interest.
