# Supplementary material for: Molecular epidemiology and genetic diversity of Listeria monocytogenes isolates from a wide variety of ready-to-eat foods and their relationship to clinical strains from listeriosis outbreaks in Chile
Source: Front Microbiol. 2015 Apr 30;6:384. doi: 10.3389/fmicb.2015.00384 (PMC4415432; doi:10.3389/fmicb.2015.00384)
Supplement: Supplementary file 1 [file Table1.DOC]

**Table S1.** PCR program used for bacterial genes amplifications.

| **Step** |  | **Temperature (°C)** | |  | **Time (min)** | |
| --- | --- | --- | --- | --- | --- | --- |
|  |  | **Virulence factors** | **Serotyping** |  | **Virulence factors** | **Serotyping** |
| Initial denaturing |  | 94 | 94 |  | 3 | 3 |
| Denaturing |  | 94 | 94 |  | 1 | 0.40 |
| Annealing |  | 57 to 62* | 53 |  | 0.45 | 1.15 |
| Extension |  | 72 | 72 |  | 0.45 | 1.15 |
| Final extension |  | 72 | 72 |  | 7 | 7 |

- Annealing temperatures for each gene are given in Table 2
